# Effects of High-Frequency Repetitive Transcranial Magnetic Stimulation on Upper Limb Dystonia in Patients With Wilson's Disease: A Randomized Controlled Trial

**DOI:** 10.3389/fneur.2021.783365

**Published:** 2021-12-14

**Authors:** Wenjie Hao, Taohua Wei, Wenming Yang, Yue Yang, Ting Cheng, Xiang Li, Wei Dong, Hailin Jiang, Nannan Qian, Han Wang, Meixia Wang

**Affiliations:** ^1^Department of Graduate, Anhui University of Chinese Medicine, Hefei, China; ^2^Department of Neurology, The First Affiliated Hospital of Anhui University of Chinese Medicine, Hefei, China; ^3^Key Laboratory of Xin'an Medicine of the Ministry of Education, Anhui University of Chinese Medicine, Hefei, China; ^4^Department of Clinical Medicine, Clinical Medicine College of Anhui Medical University, Hefei, China

**Keywords:** repetitive transcranial magnetic stimulation, Wilson's disease, upper limb myotonia, primary motor cortex, randomized controlled trial

## Abstract

**Background:** Upper limb dystonia is a frequent complication of Wilson's disease (WD). It can lead to poor quality of life and disability. Currently, no effective treatment for it exists. Therefore, we carried out a clinical trial to determine whether high frequency repetitive transcranial magnetic stimulation (rTMS) on the primary motor cortex alleviates upper limb dystonia in WD patients.

**Methods:** This study was a single-center, double-blind, randomized clinical study, included 60 WD patients with upper limb dystonia from a research base of WD in Hefei, China. Participants were randomly divided into a treatment group (TG) and a control group (CG). The TG received rTMS at 10 Hz, while the CG received sham stimulation for 7 consecutive days. Participants were assessed at baseline, after the seventh treatment session, and at 2 and 4 weeks after the seventh treatment session. The primary outcomes included patients' objective muscle tension and stiffness as measured with the MyotonPRO device. The secondary results were scores on clinical scales assessing muscle spasm and motor symptoms, which included the Modified Ashworth Scale (MAS), Unified Wilson's Disease Rating Scale (UWDRS), Burke Fahn Marsden Scale (BFM), and the Activities of Daily Living (ADL) scale.

**Results:** The analysis revealed that after 10 Hz rTMS, muscle tension (*P* < 0.01) and stiffness (*P* < 0.01) as measured by the MyotonPRO device decreased significantly in the TG compared to the CG. Moreover, clinically relevant scale scores, including the MAS (*P* < 0.01), UWDRS (*P* < 0.01), BFM (*P* < 0.01), and ADL (*P* < 0.01) were also significantly reduced.

**Conclusion:** High-frequency rTMS over the primary motor cortex may be an effective complementary and alternative therapy to alleviating upper limb dystonia in WD patients.

**Clinical Trial Registration:** http://www.chictr.org.cn/, identifier: ChiCTR2100046258.

## Introduction

Wilson's disease (WD) was first systematically described by Kinnear Wilson in 1912; it is an autosomal recessive neurological disease characterized by chronic and progressive perturbations in copper ion metabolism (1, 2). Symptoms related to the nervous system usually appear after 11 years of age. Typical symptoms include juvenile Parkinson's and dystonia; indeed, Upper limb dystonia is one of the most common symptoms (3). Currently, no recognized standard objective evaluation of muscle tension or effective treatment methods are available. WD seriously affects patients' quality of life and results in social and economic burdens. Therefore, an urgent need exists for an objective evaluation system to monitor patients' muscle tone as well as alternative therapies that relieve dystonia.

Dystonia is a neurological disorder that consists of abnormal involuntary movements or posture due to continuous or intermittent muscle contractions (4). Although its anatomical basis is controversial, recent studies have indicated that dystonia involves basal ganglia circuits, the cerebellum, and multiple cortical regions including the primary motor cortex, the premotor cortex, the supplementary motor areas, the anterior cingulate cortex, and the cerebellar cortex (5, 6). Altogether, this data has led to the network pathophysiological model hypothesis (7). Critically, related studies have reported that the motor cortex is strongly plastic when under transcranial magnetic stimulation (TMS) (8–10).

TMS is a non-invasive neuromodulation technique in which repetitive magnetic impulses are delivered to specific brain regions for short periods of time *via* stimulation coils placed over the scalp (11). TMS was invented by Barker and his colleagues in 1985 (12). Since then, it has been widely used both experimentally and clinically to study cortical function in the brains of healthy subjects as well as in the brains of patients with psychiatric and neurological disorders. The repeated magnetic pulses alter the excitability of the stimulated site while also affecting areas of the brain that are anatomically connected to it (13). Most of the TMS work has been carried out on the human primary motor cortex (M1) and has provided significant clinical and pathophysiological insights into movement disorders (14). While the evidence showing that repeated TMS (rTMS) affects the strength of synaptic connections mainly comes from experiments in the motor cortex, it is generally believed that similar effects may be observed in all regions of the neocortex (15). Notably, one large sample, double-blind, randomized study reported that high-frequency 10 Hz rTMS on M1 improved the muscle tone and stiffness of patients (16). However, to date, no studies have specifically examined the effects of high-frequency rTMS on muscle tension and muscle stiffness in WD patients.

At present, no internationally recognized objective method for detecting muscle tension exists. MyotonPRO (Myoton Muscle Diagnostics Tallinn), a digital muscle function assessment system developed by Estonia in collaboration with the European Space Center, was used in this study. The device is a non-invasive portable and reliable tonometer that can provide an objective, quantitative value of the muscle oscillation frequency (F), which in turn describes muscle tension and muscle stiffness (S) (17). The MyotonPRO device does not have a normal reference range for muscle tone, depending on race, gender, age, fat thickness, etc., but several teams are working on the project. Currently, this instrument is widely used in the fields of aerospace and sports and medical rehabilitation. Before this study, few studies have applied this instrument to detect muscle tension in WD patients.

In our study, we used an alternative therapy, rTMS, to treat WD patients with upper limb dystonia, and applied MyotonPRO device to accurately assess changes in muscle tension and stiffness. The purpose is to seek an effective alternative therapy for the treatment of WD muscle tension. We hope that our results can provide an evidence-based basis for rTMS in the treatment of dystonia in WD patients as well as provide a new objective method for evaluating muscle tension and stiffness.

## Materials and Methods

### Participants

Sixty WD patients with upper limb dystonia that were admitted to the neurology department of the First Affiliated Hospital of Anhui University of Chinese Medicine were recruited. Inclusion criteria were as follows: meet the diagnostic guidelines for WD established by the European Association for Liver Research in 2012 (18); First diagnosed; 18–35 years old; careful physical examination by two attending physicians confirmed that biceps brachii dystonia as the primary clinical presentation; and body mass index <25. The exclusion criteria were as follows: a prior use of rTMS; contraindication of rTMS; the use of drugs that can affect muscle tension, such as baclofen and benzhexol, in the 2 months before the study; using botulinum toxin treatment; presenting with anxiety or depression; and other diseases that affect muscle tension and stiffness, such as brain injury, myositis, etc.

The research program was planned in accordance with the 2010 Report Test Consolidated Criteria (CONSORT) guidelines, and the experiments were carried out following the principles of the Declaration of Helsinki. This study was approved by the Ethics Committee of the First Affiliated Hospital of Anhui University of Chinese Medicine, and all participants provided informed consent. The study has been registered at chictr.org.cn (identifier: ChiCTR2100046258).

### Study Design

This study was a single-center, double-blind, randomized controlled clinical trial. All participants were from the Department of Neurology of the First Affiliated Hospital of Anhui University of Chinese Medicine. A 1:1 proportion of patients that were hospitalized or from the clinic that met the standard of research were randomly assigned to the treatment group (TG) or control group (CG). Their group assignment was kept in a sealed envelope to ensure proper blinding. A random sequence was disclosed to those implementing the rTMS. Patients in the TG received 10 Hz rTMS stimulation, while patients in the CG received sham stimulation.

### Intervention Project

For seven consecutive days, participants in both groups (TG and CG) were treated with rTMS or sham stimulation once daily for 30 min. During the entire treatment, all participants remained seated and wore noise-canceling earplugs to eliminate peripheral noise.

For participants in the TG, the researchers connected a hand-held device to a two-phase magnetic stimulator (Magstim Rapid; Magstim, Whitland, UK). The center of the 7 cm figure-eight-shaped coil was placed over the upper limb area of the patient's scalp opposite to the upper limb with increased muscle tone (for example, a patient with increased muscle tone measured on the right upper limb would have the coil placed over the upper limb area on the left scalp). If the patient has dystonia in both upper limbs, place the coil in the upper limb area on both sides of the scalp at a 45° Angle to the sagittal plane. Each rTMS pulse was 10 Hz for 30 min for each patient. If serious adverse reactions occurred midway, the treatment was immediately stopped, and details were recorded.

For the sham stimulation in the CG, the procedure was similar to that of the TG, but the central position of the coil was at 90° to the sagittal plane. Accordingly, the coil would not induce the brain to generate electricity, and the patients would experience a subjective feeling of rTMS. In this way, not only was the control effect superior, but the blind method could also be realized.

Various measures were taken to ensure that the data collection process was blind. The operator performing the rTMS was unaware of all other assessments. All appointments were scheduled at different times so that the participants cannot communicate with each other, thereby reducing the breach of integrity. The researchers evaluated all participants at the end of the study by asking them questions regarding the method blindness.

### Clinical Assessments

**Figure 1:**
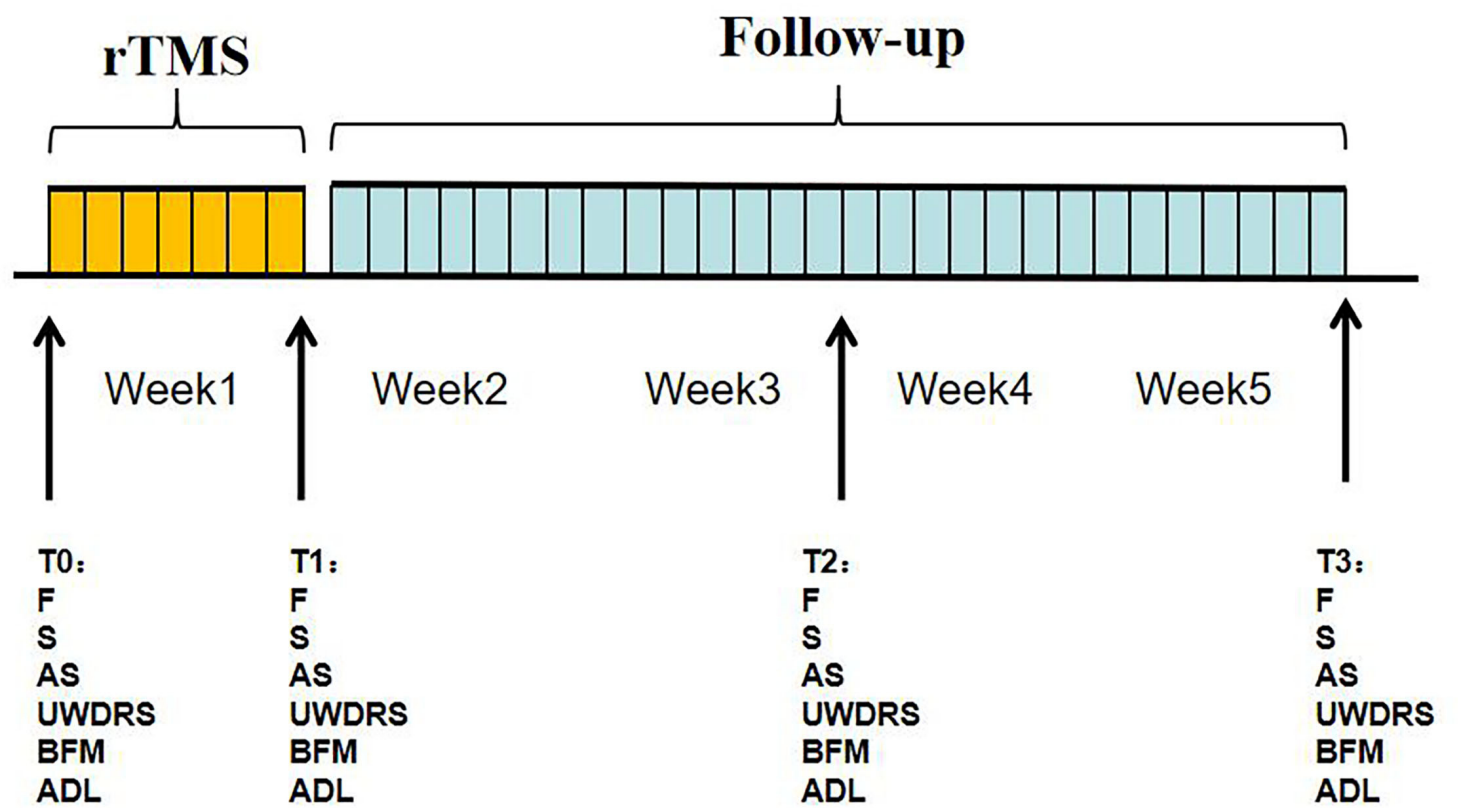
Flow chart of the study design.

Participants were clinically evaluated at enrollment (T0) and at three follow-up time points (Figure 1). The first follow-up (T1) took place after the seventh rTMS or sham stimulation, while the second (T2) and third (T3) follow-ups took place at 2 and 4 weeks, respectively, after the T1 assessment. All assessments for each participant were performed on the same day.

Muscle tone is the inherent pressure of a muscle when it is naturally relaxed without any autonomic contraction, whereas muscle stiffness is the ability to resist contraction or an external force that can deform the initial muscle shape (19, 20). The F and S values were measured using a handheld MyotonPRO device, in which the value of F is the muscle oscillation frequency, representing muscle tension, and the value of S represents muscle stiffness. All measurement processes were made in a room maintained at a temperature of 25°C. The participants were instructed to lie supine with their upper arms relaxed and forearms neutral and elbows extended. If the elbows cannot be fully extended due to muscle spasms, both sides of their forearms were supported in a neutral position. The probe was placed perpendicular to the skin surface, where it covered the biceps muscle midway between the medial condyle of the humerus and the acromion. Brief mechanical pulses were given, and the oscillations (which were dampened due to the soft tissue) were recorded. Five measurements were taken, and their average was calculated.

Clinically relevant scales, including the Muscle Spasticity Score (Modified Ashworth Scale, MAS), Unified Wilson's Disease Rating Scale (UWDRS), Dystonia Scale (Burke Fahn Marsden Scale, BFM), and Activities of Daily Living (ADL) were assessed by trained psychiatrists who had no knowledge of the patient's grouping. In all patients, adverse events were screened at the T0, T1, T2, and T3 timepoints.

### Statistical Analysis

Statistical analyses were performed using SPSS 22.0 statistical software. Numerical data are expressed as mean ± standard deviation (mean ± SD), and compared using *t*-tests for normally distributed data and the Rank Sum Test for data that followed a non-normal distribution. The chi-squared test was used to compare categorical data. Correlation analysis was conducted by *Pearson* correlation analysis. *P* < 0.01 was considered statistically significant.

## Results

### Baseline Characteristics

**Figure 2:**
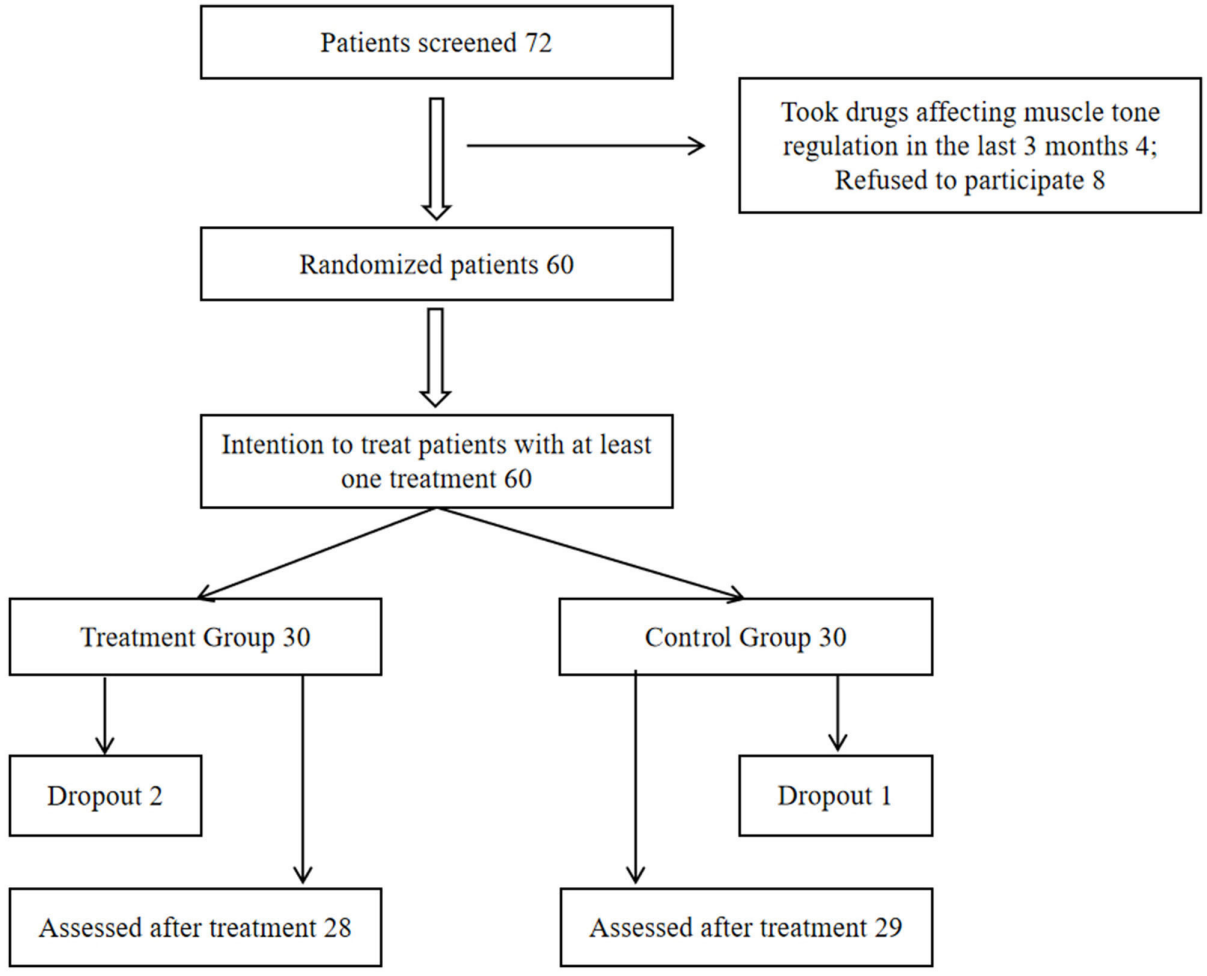
Flow chart of the study design.

Seventy-two WD patients aged 18–35 years met the inclusion criteria. Four patients were excluded on account of previous treatments with drugs affecting muscle tone, and eight patients refused to participate. Among the remaining 60 patients, three of them did not complete the study due to non-compliance with the experiment schedule. Altogether, 57 patients completed the study and were included in the analysis. The flow chart of the research design is shown in Figure 2. The demographic and baseline clinical (F, S, MAS, UWDRS, BFM, and ADL scores) characteristics are summarized in Table 1. They were similar between the TG and CG (*P* > 0.05).

**Table 1:** Demographic and baseline clinical characteristics of both study groups.

**Characteristics**	**TG (*N* = 28)**	**CG (*N* = 29)**	***P***
Subjects (male/female)	30 (16/14)	30 (15/15)	
Dropped out	2	1	
Completed (male/female)	28 (15/14)	29 (14/15)	
Age (years)	22.21 ± 3.17	22.45 ± 4.69	0.827
Course of disease (month)	33.79 ± 4.69	34.90 ± 4.55	0.368
BMI (kg/m^2^)	19.26 ± 3.06	18.01 ± 3.05	0.128
Under a chelating therapy (*n*)	0	0	1
Antidystonic drugs used (*n*)	0	0	1
F (Hz)	16.05 ± 1.96	15.34 ± 1.94	0.176
S (N/m)	231.12 ± 15.35	236.86 ± 15.98	0.172
MAS	3.43 ± 0.69	3.41 ± 0.68	0.936
UWDRS	54.57 ± 6.33	53.90 ± 5.22	0.662
BFM	64.68 ± 12.37	64.86 ± 10.24	0.952
ADL	28.32 ± 3.72	28.00 ± 5.90	0.807

*Means and SD are shown for continuous variables*.

*TG, treatment group; CG, control group; BMI, body mass index; F, oscillation frequency; S, stiffness; MAS, Modified Ashworth Scale; UWDRS, Unified Wilson's Disease Rating Scale; BFM, Burke Fahn Marsden Scale; ADL, Activities of Daily Living*.

### Clinical Efficacy: Primary Outcomes (F and S)

**Figure 3:**
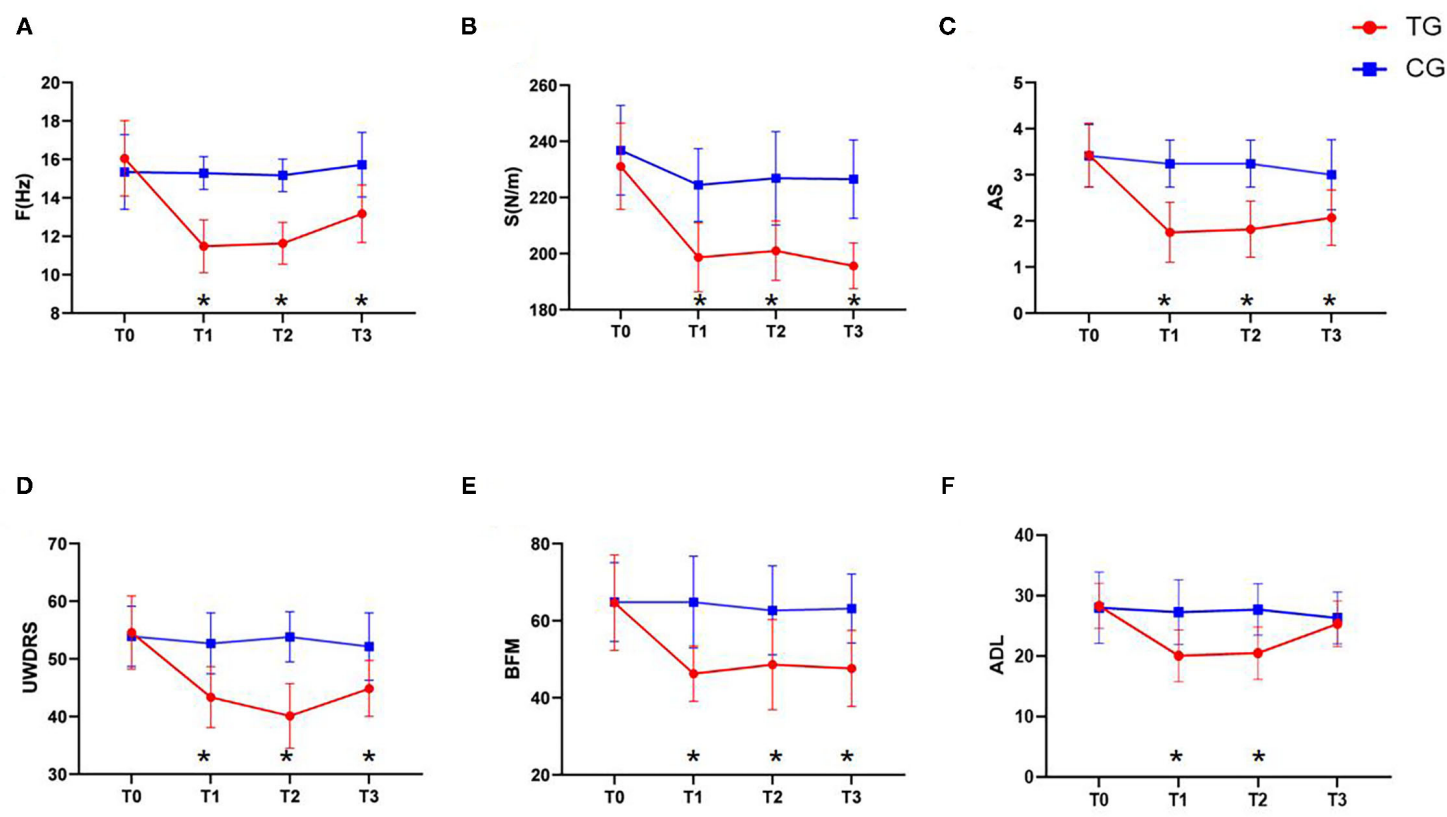
Changes in clinical efficacy after rTMS. **(A)** The change of the F value. **(B)** The change of the S value. **(C)** The change of the UWDRS scores. **(D)** The change of the AS scores. **(E)** The change of the BFM scores. **(F)** The change of the ADL scores. Red: TG; blue: CG. Error bars represent SD; *represent significant difference between TG and CG at the same time point.

As shown in Figure 3, our analysis of the F and S values of the brachii as measured by the MyotonPRO device revealed that, at time points T1 (*P* < 0.01), T2 (*P* < 0.01), and T3 (*P* < 0.01), these values in the TG were significantly lower than those in the CG (Figures 3A,B). These results indicate that rTMS treatment can effectively improve the muscle tone and stiffness of WD patients with increased upper limb dystonia.

### Clinical Efficacy: Secondary Outcomes (Related to the Scale)

As shown in Figure 3, our analysis of clinical related scales demonstrated that MAS, UWDRS, and BFM scores in the TG were significantly lower than those in the CG at the T1 (*P* < 0.01), T2 (*P* < 0.01), and T3 (*P* < 0.01) time-points. In contrast, the ADL scores in the TG were significantly lower than those in the CG at T1 (*P* < 0.01) and T2 (*P* < 0.01), but the difference was not significant at T3 (*P* = 0.376) (Figures 3C–F). These results suggest that rTMS treatment can effectively improve the muscle spasms and changes in muscle tone in WD patients with increased upper limb dystonia and thereby improve their daily living.

### Correlation Analysis

As shown in Table 2. We analyzed the correlation between myotonpro instrument parameters and the scores of related scales. The results showed that the F value was significantly positively correlated with the S value, the scores of MAS, UWDRS, BFM and ADL scales (*P* < 0.01).

**Table 2:** *Pearson* correlation analysis between F value measured by MyotonPRO device with the S value, the score of MAS, UWDRS, BFM, and ADL scale.

**Outcome**	***r***	***P***
S	0.576	<0.01
MAS	0.571	<0.01
UWDRS	0.512	<0.01
BFM	0.506	<0.01
ADL	0.342	<0.01

### Adverse Events

The incidence of adverse events between the two groups (TG and CG) were similar, and no serious adverse events occurred. The adverse events in this study were: dizziness 5–8 min (three cases in each group), tinnitus 10–20 min (three cases in TG, two cases in CG), and headache 0.5–1 h (1 case in each group).

## Discussion

This study is the first controlled clinical trial that used the MyotonPRO to objectively evaluate dystonia and the efficacy of rTMS in the treatment of upper limb dystonia in WD patients. In this study, we found that high-frequency rTMS targeting M1 alleviated biceps brachii dystonic disorders in WD patients and significantly improved the biomechanical properties (F/S values) of the bicep muscles as well as MAS, UWDRS, BFM, and ADL scores. These results suggest that high-frequency rTMS targeting the M1 can reduce muscle tone and muscle spasms in WD patients as well as significantly improve their quality of life.

The standard treatment for WD is oral chelating substances (e.g., D-penicillamine and trientine) to enhance urinary copper excretion. Unfortunately, not all neurological symptoms respond equally well to this therapy, and sometimes D-penicillamine can even make them worse (21). Neurological symptoms may persist or even progress after treatment with chelates in up to 65% of WD patients. Neurological WD patients may develop Parkinson's symptoms, such as slow movement, rigidity, weakness, gait and postural disorders, dysarthria and dysphagia, at which time symptomatic treatment is required (22, 23). Anticholinergic drugs, baclofen, levodopa, trihexaphenyl, benzodiazepine, carbamazepine/oxamamazepine, and Botulinum neurotoxin have been recommended for symptomatic treatment of WD (24, 25). Multisegmental or systemic dystonia may be treated with the above recommended oral medications, while focal dystonia may be treated with Botulinum toxin (BTX) injections (26, 27). However, cell and animal experiments suggest that BTX may be less effective in Wilson's disease (28). Hefter et al. (29) reported that out of 156 WD patients regularly seen at the outpatient department of Dusseldorf University Hospital (Germany), only 6 patients had received botulinum neurotoxin A (BoNT/A) in the past 5 years. BoNT/A injection therapy is A rare (<4%) for symptomatic treatment of WD, which is needed only in exceptional circumstances and is usually used only temporarily. Holscher reported that 4 cases of dystonia were treated with anticholinergic drugs with good efficacy and 1 case was ineffective (30). Anticholinergic drugs can cause adverse events, mostly in older patients, while younger patients can tolerate higher doses, which is typical in WD cases (25). Poujois et al. (31) reported one patient with a severe dysarthria of WD who experienced a real and prolonged improvement of her voice after treatment by zolpidem. Relevant studies have shown that Chinese herbal medicine may alleviate neurological symptoms in WD animal models (32). There is no specific consensus on the treatment of WD neurological dysfunction (33). Dystonia can be treated with neurosurgery when medication fails, including deep-brain stimulation of globus pallidus internus, pallidotomy, or thalamotomy (34).

The core anatomy and dysfunction of WD is located in the basal ganglia, which is too deep to be easily stimulated by normal TMS coils. However, the basal ganglia are well-known to be part of a set of parallel closed circuits (basal ganglia-thalamic- cortical-basal ganglia) that originate in the cerebral cortex, cross the thalamus, and return to their respective frontal lobe origin (35). Notably, some WD patients have cerebral cortex damage (36). Thus, even though the direct effects of the TMS may be limited to the cerebral cortex, stimulation of this cortical region, if it is part of the basal ganglia circuitry, can affect the activity of these circuits and produce clinical effects. Confirming this idea, functional magnetic resonance imaging studies in humans have demonstrated that TMS of the presupplementary motor area and the left dorsal premotor cortex can increase the amount of signal in the striatum and thalamus (37). Moreover, positron emission tomography imaging has revealed that TMS can produce dopamine in the striatum (38). Although few studies have been carried out in WD models, this idea has been confirmed in animal studies of related movement disorders such as Parkinson's disease (PD). By interacting with a node in a complex circuit, the activity of a node some distance away can be changed. In an animal model of PD, optogenetic studies have indicated that the stimulation of afferent from the cortex to the subthalamic nucleus ameliorates hypokinesia and bradykinesia and leads to a broad increase in glutamate activity in the thalamus and other brain regions (16).

The mechanism by which rTMS alleviates WD dystonia remains unclear. Low or high frequency rTMS can induce lasting inhibitory or excitatory effects. Study regimens with extremely long stimulation times have positive or negative effects on cortical excitability and are associated with the prevention of cell death, brain-derived neurotrophic factor regulation, and γ-aminobutyric acid interneuron-mediated inhibition (39). rTMS has been used as a therapeutic strategy to modulate the brain regions involved in dystonia. Our results demonstrate that rTMS targeting M1 improves muscle tone and stiffness in WD patients and improves their ability to perform daily tasks, which is consistent with the findings of other related diseases (40). While a study has reported that low-frequency rTMS did not significantly improve the muscle tone of WD patients, these negative results could be due to the study's small sample size and short treatment time (41). Accordingly, perhaps only high frequency rTMS can obtain positive results for WD dystonia. However, note that results between studies can vary due to different rTMS frequencies and different locations of stimulation (42). Regardless, we hope that the effect of rTMS on WD dystonia can be systematically and comprehensively studied in the future.

In conclusion, TMS may affect the strength of synaptic connections in the human cortex and improve muscle tone. The evidence comes mainly from basic and clinical trials of the motor cortex, and it is widely believed that similar effects can be observed in all regions of the neocortex. For diseases of the basal ganglia, direct changes in the functioning of cortical areas *via* TMS may have secondary effects on the connective structure within the cortical ring of the basal ganglia. This effect is the rationale for attempting its use in the treatment of basal ganglia disease (11). Evidence suggests that rTMS is a useful treatment option for WD dystonia.

This study has some limitations that need to be considered. First, participants in this study were recruited from one center, with a small number of cases. Accordingly, the research results may not be highly generalizable. We hope to cooperate with other international hospitals in the future to conduct a multi-center, large-sample study. Second, we used the classical scheme of positioning the coil 90° as a sham strategy. Although several other ways to induce spurious stimuli exist, it has been demonstrated that turning the coil 90°, while not completely avoiding the stimulus, can induce much lower voltages than active rTMS (43). This scheme has been widely used in previous studies (44). Third, in clinical practice, WD patients can have various manifestations of dystonia. In this study, only patients with increased biceps muscle tension were selected. We hope to study more types of dystonia in WD patients in future studies. Finally, we only studied the short-term improvement of rTMS; the trial was evaluated over a period of 1 month, so our results may not reflect its long-term effects.

## Conclusions

Our results suggest that 10 Hz rTMS can reduce the symptoms of biceps dystonia in WD patients in a short period of time and thereby improve their quality of life. Thus, our protocol can be used as a complementary or replacement therapy to relieve muscle spasm in WD patients.

## Data Availability Statement

The original contributions presented in the study are included in the article/supplementary material, further inquiries can be directed to the corresponding author.

## Ethics Statement

The studies involving human participants were reviewed and approved by the First Affiliated Hospital of Anhui University of Chinese Medicine. The patients/participants provided their written informed consent to participate in this study.

## Author Contributions

WH and WY conceived and designed the study. WH, TW, YY, and TC participated in the recruitment of participants, data analysis, and management. XL, WD, HJ, and NQ implemented the treatment and collected the data. HW and MW contributed to the study conception, evaluation, study execution, and manuscript preparation. WH drafted the manuscript, while WY revised the manuscript. All authors read and approved the final manuscript.

## Funding

This work was supported from the National Administration of Traditional Chinese Medicine: 2019 Project of building evidence based practice capacity for TCM (No. 2019XZZX-NB001), the National Natural Science Foundation of China (Grant No. 81973825), the Anhui Provincial Natural Science Foundation of China (Grant No. 2108085QH367), the Open Fund Project of Key Laboratory of Xin'An Medicine of Ministry of Education (No. 2020xayx12), and the University Synergy Innovation Program of Anhui Province (No. GXXT-2020-025).

## Conflict of Interest

The authors declare that the research was conducted in the absence of any commercial or financial relationships that could be construed as a potential conflict of interest.

## Publisher's Note

All claims expressed in this article are solely those of the authors and do not necessarily represent those of their affiliated organizations, or those of the publisher, the editors and the reviewers. Any product that may be evaluated in this article, or claim that may be made by its manufacturer, is not guaranteed or endorsed by the publisher.
